# Supplementary material for: Peroxygenase-Catalyzed Selective Synthesis of Calcitriol Starting from Alfacalcidol
Source: Antioxidants (Basel). 2022 May 25;11(6):1044. doi: 10.3390/antiox11061044 (PMC9220053; doi:10.3390/antiox11061044)
Supplement: Supplementary file 1 [file antioxidants-11-01044-s001.zip › antioxidants-1687999-supplementary.pdf]

Antioxidants

Supporting Information

Peroxygenase-Catalyzed Selective Synthesis of Calcitriol Starting from Alfacalcidol

Yuanying Li [1,2], Pengpeng Zhang [1,2], Zhoutong Sun [1,2], Huanhuan Li[1,3], Ran Ge [1,2], Xiang Sheng [1,2], and Wuyuan Zhang[1,2]*

[1] Tianjin Institute of Industrial Biotechnology, Chinese Academy of Sciences, Tianjin 300308, China; liyy@tib.cas.cn (Y.L.); zhangpp@tib.cas.cn (P.Z.); sunzht@tib.cas.cn (Z.S.); li-huan@stu.xjtu.edu.cn (H.L.); ger@tib.cas.cn (R.G.); shengx@tib.cas.cn (X.S.); zhangwy@tib.cas.cn (W.Z.)

[2] National Center of Technology Innovation for Synthetic Biology, Tianjin 300308, China

[3] School of Chemical Engineering and Technology, Xi'an Jiaotong University, Xi'an

* Correspondence: zhangwy@tib.cas.cn; Tel.: +86 22-8486-6462

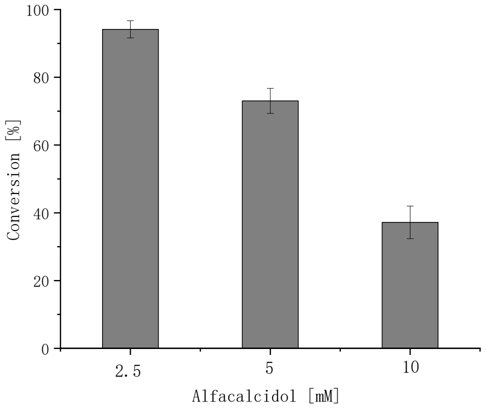

Figure S1. *Aae*UPO (Pada-I)-catalyzed oxyfuntionalization of alfacalcidol to calcitriol (path I) at various substrate loadings. Conditions: [*Aae*UPO] = 2.5 μM, [substrate] = 2.5-10 mM, NaPi buffer (50 mM, pH 6), 40% (v/v) acetone, [H_2O_2] = 1.5 mM h^{-1}, 8 h, 25 °C, 1 mL.

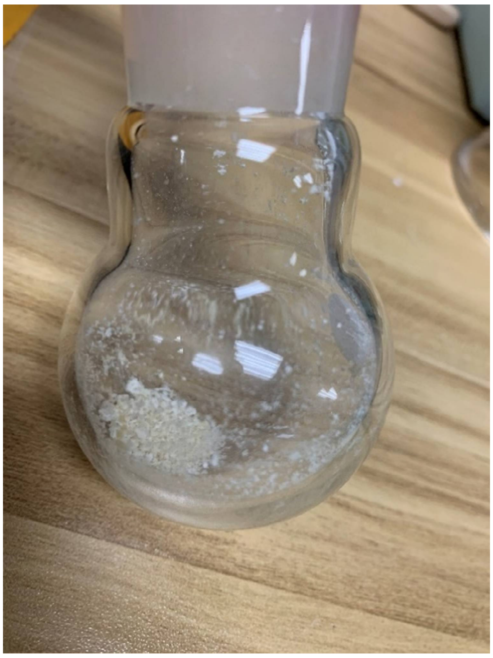

Figure S2. Image of the isolated product calcitriol (150 mg) obtained from a semi-preparative scale synthesis.

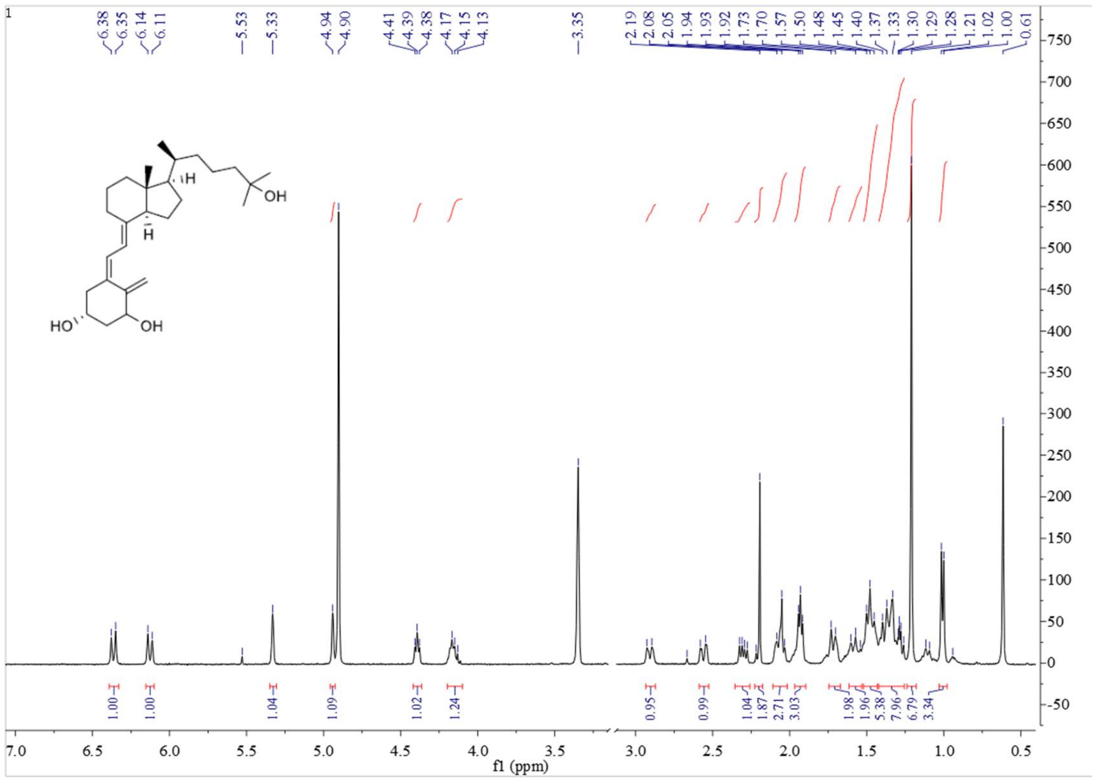

Figure S3. ^{1}H NMR of the isolated product calcitriol in CD_3OD: (400 MHz, δ, ppm: 6.36-6.39 (d, 1H), 6.00-6.03 (d, 1H), 5.32 (s, 1H), 5.00 (s, 1H), 4.41- 4.44 (m, 1H), 4.22- 4.24 (m, 1H), 1.00-1.60 (m, 28H), 0.90- 0.95 (m, 3H), 0.75- 0.92 (m, 4H), 0.54 (s, 3H)).

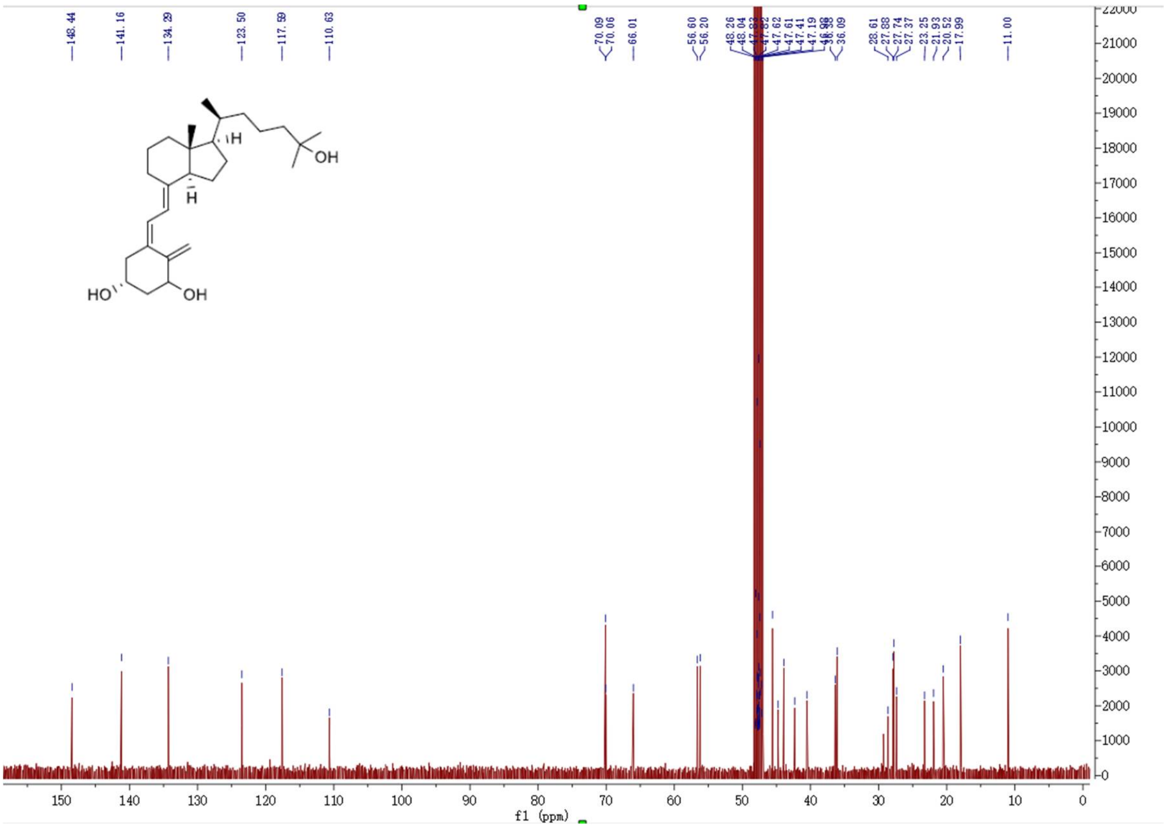

Figure S4. ^{13}C NMR of the isolated product calcitriol in CD_3OD: (400 MHz, δ, ppm: 148.44, 141.16, 134.29, 123.50, 117.59, 110.63, 70.09, 70.06, 66.01, 56.60, 56.20, 45.59, 44.76, 43.91, 42.32, 40.54, 36.38, 36.09, 28.61, 27.88, 27.74, 27.37, 23.25, 21.93, 20.52. 17.99, 11.00).
